# Specialized Knowledge Representation and the Parameterization of Context

**DOI:** 10.3389/fpsyg.2016.00196

**Published:** 2016-02-23

**Authors:** Pamela Faber, Pilar León-Araúz

**Affiliations:** Department of Translation and Interpreting, University of GranadaGranada, Spain

**Keywords:** context parameters, specialized knowledge, terminology, terminological knowledge bases

## Abstract

Though instrumental in numerous disciplines, context has no universally accepted definition. In specialized knowledge resources it is timely and necessary to parameterize context with a view to more effectively facilitating knowledge representation, understanding, and acquisition, the main aims of terminological knowledge bases. This entails distinguishing different types of context as well as how they interact with each other. This is not a simple objective to achieve despite the fact that specialized discourse does not have as many contextual variables as those in general language (i.e., figurative meaning, irony, etc.). Even in specialized text, context is an extremely complex concept. In fact, contextual information can be specified in terms of scope or according to the type of information conveyed. It can be a textual excerpt or a whole document; a pragmatic convention or a whole culture; a concrete situation or a prototypical scenario. Although these versions of context are useful for the users of terminological resources, such resources rarely support context modeling. In this paper, we propose a taxonomy of context primarily based on scope (local and global) and further divided into syntactic, semantic, and pragmatic facets. These facets cover the specification of different types of terminological information, such as predicate-argument structure, collocations, semantic relations, term variants, grammatical and lexical cohesion, communicative situations, subject fields, and cultures.

## Introduction

According to Akman and Surav (1997) and Akman (2000), the denotation of *context* has become murkier as its uses have spread out in many directions to the extent that it has become a sort of ‘conceptual garbage can.’ For this reason, efforts are currently being made to parameterize and generally make sense of *context* and all that it implies. However, though instrumental in numerous disciplines, context has no universally accepted definition, because it can point to many different things. In the same way as the definition of any word, the definition of context can also vary depending on the field of application, such as Linguistics, Cognitive Science, or Computer Science (Bazire and Brézillon, 2005).

Specialized knowledge is related to all of these three areas in the sense that (1) it is shared and disseminated through linguistic communicative acts (journal articles, conferences, etc.); (2) it is processed and acquired in the mind; and (3) it may be subjected to formalization. Therefore, the parameterization of context for specialized knowledge representation should be approached from a multidisciplinary perspective.

Specialized knowledge can be represented in a variety of formats (i.e., ontologies, vocabularies, thesauri, controlled languages, databases, etc.) that may or may not support context, because knowledge resources are conceived for very different purposes (i.e., classification, reasoning, knowledge acquisition, standardization, harmonization, information retrieval, machine, or human translation, etc.). More specifically, terminological knowledge bases (TKBs) generally describe the concepts and terms of specialized knowledge domains for users with linguistic and/or cognitive needs. TKB users are most often human (e.g., translators, experts, technical writers), but computer applications can also benefit from terminological resources when it comes to automatically interpreting and/or producing specialized texts. Even though TKBs usually provide conceptual representations based on some sort of knowledge modeling mechanism, they rarely support context modeling. In other words, very few provide controlled partial information concerning conceptual entities by viewing them from different viewpoints or situations. This can be a problem because the meaning, designation, collocates, and location of a concept within a knowledge configuration or linguistic structure often vary, depending on context.

Contextual information must thus be included in a TKB that aspires to being a knowledge representation resource. In this regard, it is timely and necessary to parameterize context in specialized knowledge domains with a view to more effectively facilitating knowledge representation, understanding, and acquisition. Nevertheless, matters are further complicated by the fact that context itself is a complex, multidimensional concept. Reasons for its conceptual fuzziness include the following: (i) there are various types of contexts; (ii) many types of data can be extracted from context analysis; (iii) contexts can also be used for a wide range of different purposes.

Contextual information can be specified in terms of scope (local vs. global) or according to the type of information conveyed (syntactic, semantic, and pragmatic variables). As reflected in corpus analysis, when context is mentioned in a text, it is metaphorically conceived as a container or a bounded space, since an utterance can be “in context” or “out of context.” Context also *frames* or *surrounds* the utterance or object of analysis. In this sense, context bears a resemblance to Fauconnier’s (1985, 1997) mental spaces since the location of an utterance in this bounded space or container is what makes it meaningful. As a relational construct in texts, context helps to anchor linguistic designations to objective reality by providing background information, situating objects and processes, and explicitly relating them to each other as well as to the agents that manipulate them and act on them. It is thus a constraining factor that drives understanding. In other words, as stated by Leech (1981), the specification of context (whether linguistic or non-linguistic) has the effect of narrowing down the communicative possibilities of the message as it exists in abstraction from context.

The remainder of this paper is organized as follows. The Section “What is Context?” reviews the notion of context as found in the literature of different areas. In Section “Context and Terminology,” context representation is described with regards to terminology and specialized knowledge. The Section “Context Parameters” proposes a taxonomy of context parameters from a local to a global scope further divided into syntactic, semantic, and pragmatic facets. These facets cover the specification of different types of contextually relevant terminological information, such as predicate-argument structure, collocations, semantic relations, term variants, grammatical, and lexical cohesion, communicative situations, subject fields, and cultures. The examples given are drawn from the domain of environmental science based on the experience acquired while building EcoLexicon (ecolexicon.ugr.es), an environmental multilingual TKB. The Section “Conclusion and Future Work” provides the conclusions derived from the parameterization of context for specialized knowledge representation.

## What is Context?

Research communities envision context differently since they conceive it in relation to different entities. Thus, context may be the parts of discourse surrounding a word, sentence, or passage, also known as co-text (Textual Linguistics), the set of situational elements where the object being processed is included (Cognitive Psychology), or that which surrounds and gives meaning to something else (Computer Science).

In Linguistics, context has long been regarded as an essential factor in the interpretation of linguistic utterances. It plays an important role in different tasks, such as meaning construction, inference, variation, modulation, sense disambiguation, etc. Quite often co-textual elements are sufficient to resolve ambiguity, but sometimes other context types also come into play.

Apart from the co-text sense, context in Linguistics is also mentioned in relation to pragmatic and cognitive notions, such as speech acts (Austin, 1962; Searle, 1969), conventions (Gadamer, 1995), maxims (Grice, 1975), framing (Goffman, 1974), common ground (Clark, 1996), and mutual manifestness (Sperber and Wilson, 1986, 1990), which refers to what one is capable of inferring or perceiving even if one has not done so as yet. The sum of these shared assumptions constitutes the cognitive environment of a group of individuals, which provides the foundation for successful communication (Yus, 2006).

These notions are related to sociocultural factors accounting for broader contextual variables, such as communicative settings, cultures, or world knowledge. Evidently, context has also been extensively studied in discourse studies, where it has been defined as the totality of conditions under which discourse is being produced, circulated and interpreted (Blommaert, 2005, p. 251). In the same area, Van Dijk (2005, p. 237) gives an even wider view by dividing context in different dimensions, namely, the cognitive, social, political, cultural, and historical environments of discourse.

In Cognitive Science, since the emergence of situated cognition, background situations have also become an essential element in the analysis of context. This has had an impact on cognitive linguistics, where meaning is thought to be based mostly on situational context and constructed on-line (Croft and Cruse, 2004; Evans and Green, 2006). Meaning thus does not exist without context. For example, the theory of situated cognition argues that knowledge is situated, and is partly a product of the activity, context and culture in which it is developed and used (Brown et al., 1989, p. 32). Clancey (1994) adds that the situated aspect of cognition is that the world is not given as objective forms. Rather, what we perceive as properties and events is constructed according to the context.

Elman (2009, p. 572) highlights the importance of context in language comprehension and asserts that the meaning of a word is rooted in our knowledge of both the material and social world. Therefore, the meaning of a word is never ‘out of context’ even when we are not aware of what this context is. He also highlights the importance of larger knowledge structures: “events play a major role in organizing our experience. Event knowledge is used to derive inference, to access memory, and affect the categories we construct. An event may be defined as a set of participants, activities, and outcomes that are bound together by causal relatedness.” Consequently, all lexical units, apart from their micro-context in discourse, need to be understood within the context of a larger event.

According to Yeh and Barsalou (2006, p. 350), knowledge of a larger event or situation restricts the entities and events likely to occur in it. Conversely, knowledge of current entities and events constrains the event or situation likely to be unfolding. Context thus plays a crucial role in knowledge understanding and acquisition since it can trigger one meaning while inhibiting another.

Cognitive processing necessarily includes linking an utterance or object to the right context, something that the human brain does with relative ease. In this sense, according to Flowerdew (2014), speakers and writers are remarkably adept at knowing which features of context to rely on to make their utterances meaningful, and listeners and readers are equally adept at contextualizing what they read or hear in order to understand it. However, what is not so easy is to agree on how to characterize context types and describe how they interact with each other. In fact, context was for a long time omitted in linguistic accounts because it was considered to be too chaotic and idiosyncratic, to be systematically characterized (Ervin-Tripp, 1996, p. 35).

Despite the evident challenge, the benefits of formalizing context are well known in computing. Computer Science has been dealing with context as a formal object –although more limited in scope– for some time now since McCarthy (1987, 1993), who stated that there is simply no most general context where all the stated axioms always hold and are meaningful.

From a computational perspective, contexts are useful for putting together a set of related axioms. In this way, contexts are used as a means for referring to a group of related assertions about which something can be said (Guha, 1991). However, the notion of context in computer science has two sides (Brézillon, 2005). Firstly, there is the cognitive science view, where context is used to model interactions and situations in a world of infinite breadth and human dimension, which is the key for extracting a model. Secondly, there is the engineering view, where context is useful in representing and reasoning about a restricted state space within which a problem can be solved. Since context, knowledge and reasoning are closely intertwined (Brézillon, 2005), the main aims of artificial intelligence with regards to the formalization of context seem obvious: (i) performing automatic inferences and reasoning (Guha, 1991; Lenat, 1995); (ii) identifying relational constraints for human–computer interaction and context-aware applications (Dey, 2001); (iii) improving automatic information retrieval, resolving ambiguities in natural language processing (NLP), etc. Also relevant to the parameterization of context is the concept of explanatory coherence (Thagard, 1989), which formalizes and computes coherence as a constraint satisfaction problem (Thagard and Verbeurgt, 1998).

Although some of these applications go beyond the scope of this proposal, there are others that could benefit from the systematization of context features in specialized knowledge resources, especially those related to NLP and domain ontologies. More specifically, with regards to knowledge representation and reasoning, context is needed to derive new knowledge from what is already known.

However, context is more than a set of previously specified discrete variables that have an impact on the knowledge of a language and a person’s ability to use it. Context and language are considered to be in a mutually reflexive relationship, such that language shapes context as much as context shapes language (House, 2006).

## Context and Terminology

As is well known, Terminology is the study of how specialized knowledge concepts are structured, described, and designated in one or various languages within a specialized domain. One of the practical tasks in Terminology is the design and creation of terminological resources so that users, whether human or artificial, can effectively access concepts and associated information in order to understand, acquire, or produce specialized knowledge.

Although the tendency in the General Theory of Terminology (Wüster, 1979) was initially to disregard context and contextual variables as well as the terminological variation that they produce, it soon became apparent that specialized terms are lexical items that are used in communicative contexts (Sager, 1990; Cabré, 1999), and that these contexts can affect their potential meaning. In fact, specialized knowledge units or terms acquire their meaning in context, more specifically, within a frame including a semantic and pragmatic background (Reimerink et al., 2010).

Nevertheless, contextual information is rarely found in specialized knowledge resources. As pointed out by Bowker (2011), most term banks present terms out of context, or in only a single context. A possible reason is the widespread belief that terms in the same field never have more than one meaning and thus have a one-to-one relation with the object or process designated. However, terms and concepts are dynamic and context-sensitive. For instance, concepts may be recategorized so as to constrain their relational behavior, and terms may show several types of variants with different cognitive, semantic, and usage consequences (León-Araúz and Faber, 2014), (see examples in Sections “Local Pragmatic Contexts” and “Global Pragmatic Contexts”).

User understanding of an entity or group of entities depends on having access to the necessary information to activate the right frame or knowledge structure in which the word or term should be processed. In turn, the effective production of a specialized utterance also depends on the user having access to the combinatorial potential of the terms involved. When a terminological resource includes multilingual correspondences, contextual information becomes even more crucial because of the lack of isomorphism between languages and cultures.

Generally speaking, even when contextual information is included in the concept or term entries of knowledge resources, it is not inserted in a systematic way since there is no consensus of opinion on the exact nature of context. The most common form of context found in terminological resources is a textual excerpt where terms are shown in real use, whether such contexts are in the form of KWIC (Key Word in Context) concordances or longer full-sentence segments. These can be useful to enhance both linguistic and cognitive user needs since they can provide valuable information regarding the collocational behavior of the terms and/or the relational behavior of the concepts activated. However, this is only a small fraction of what context representation should be.

Dubuc and Lauriston (1997, pp. 81–83) were among the first to underline the importance of context in Terminology: “Contexts are important to terminology with respect to the relationship of a term with its field of application. The context embodies the discourse bearing the term […]. It is the presence of conceptual features relevant to the term that determines the extent of the context.” Despite the fact that their interest in context was restricted to evidence of the term being used in the specialized field and the conceptual content associated with the term, this was still a relatively new assertion for the time. They classified contextual excerpts as associative, explicative, or defining, depending on the quantity and quality of concept descriptors obtained. This seminal study focused on context as a way of enhancing the reader’s mental image of a concept.

Pearson (1998) goes somewhat further and explains why context is a great deal more than a text excerpt included in a term entry for purposes of knowledge acquisition. She affirms that the only way of determining what a term is and whether language is behaving ‘terminologically’ is by examining context. A context thus reflects a certain communicative setting, which is the most important factor that shows whether a given lexical unit is being used as a term or as a general language word. Finally, she also highlights the usefulness of metalanguage patterns retrieved from corpora in the formulation of terminological definitions (ibid: 191–203). This was subsequently complemented by Meyer (2001), who introduced the notion of *knowledge-rich context*.

Not surprisingly, in the last 15 years, context has become an important focus in Terminology research and its uses have multiplied accordingly. In its co-text sense, it is currently a primary data source for elaborating and constraining the scope of meaning definitions. It has thus become a rich source of complementary conceptual information, linguistic usage, and knowledge representation, *inter alia*. Nevertheless, as observed in Section “What is Context?,” context encompasses much more. Other than static text-based usage examples, context representation in Terminology should also cover background situations, cultures, communicative settings, etc.

The vital role of specifying context and of embedding specialized concepts in situations has been highlighted as a way of enriching conceptual representations in TKBs. According to Meyer et al. (1992), TKBs should reflect conceptual structures similarly to how concepts are related in the human mind. Similarly, Faber (2011) states that the organization of semantic information in the brain should underlie any theoretical assumption concerning the retrieval and acquisition of specialized knowledge concepts as well as the design of specialized knowledge resources.

For example, in an fMRI study of expert-novice differences in the identification of geological field instruments, Faber et al. (2014b) found that in contrast to novices, experts activated the bilateral precuneus, posterior cingulate, and insula, three regions previously implicated in mental imagery, episodic memory, and context representation. In addition, the importance of visual scene generation was reinforced by brain activation in the parahippocampal gyrus, which encodes meaningful contextual associations.

In Frame-Based Terminology (FBT; Faber et al., 2005, 2006, 2007; Faber, 2011, 2012, 2015), specialized knowledge units are only understood with reference to their underlying conceptual frame, whose elements are selected according to context. Context determines the activation of previously stored knowledge and the formation of new categories (Croft and Cruse, 2004 p. 75). In this sense, Barsalou (1983, 1991) found that conceptual categories can be created in an *ad hoc* goal-derived way, which indicates that context determines the conceptual organization underlying a concrete situation. Since categorization itself is a dynamic context-dependent process, the representation and acquisition of specialized knowledge should certainly focus on contextual variation (León-Araúz et al., 2013).

For this reason, one of the keys to the enhancement of specialized knowledge resources lies in parameterizing contextual information. This entails distinguishing different types of context, their scope and facets as well as how they interact with each other. This is not a simple objective to achieve despite the fact that specialized discourse does not have as many contextual variables as those in general language (e.g., figurative meaning, irony, etc.).

A solid theory of context and context types would be a timely contribution to lexical semantic research which would have repercussions in a wide range of fields. A principled set of context modeling parameters would facilitate knowledge acquisition and understanding. Such resources would ideally allow non-experts to understand a given domain by focusing on and capturing essential knowledge. However, they would also benefit diverse applications in NLP and in the Multilingual Semantic Web (MSW; León-Araúz and Faber, 2014). The MSW is envisioned as an information space where language-independent knowledge would be accessible across different natural languages. This entails the improvement of many NLP techniques related to both comprehension and production, such as word sense disambiguation, cross-lingual mappings, or question answering –always depending on general language resources such as WordNet. Thus, for the web to be truly semantic and multilingual, different NLP tools and techniques need to rely on high-quality multilingual resources –whether general or specialized– that account for the representation of context, a major barrier to successful communication.

## Context Parameters

Many authors have proposed the characterization of context types, based on a wide range of different criteria. In Cognitive Linguistics, Evans and Green (2006, p. 21) underline the importance of different types of context in the modulation of any given instance of a lexical item as it occurs in a particular usage event. Broad context types mentioned are the following: (1) encyclopedic context (information accessed within a network of knowledge); (2) sentential context (utterance meaning); (3) prosodic context (intonation pattern); (4) situational context (physical location where the text is emitted); and (5) interpersonal context (relationship holding between text sender and receiver). Most other approaches give a more binary vision of context. For instance, Harris (1988) proposes *world knowledge* vs. *language knowledge*, whereas Halliday (1989) makes the distinction, *context of situation* vs. *context of culture*. This duality can also be found in the distinction between context and co-text.

In reference to specialized knowledge units, the primary division of context is based on scope, since contexts can be either local or global. Context may be a few words on either side of a term (He et al., 2010), the sentence or paragraph in which it is appears (Soricut and Marcu, 2003), a set of documents containing it (Cilibrasi and Vitanyi, 2007), a communicative act, or even a whole culture. According to Akman and Bazzanella (2003, p. 325), an adequate multi-modal coding of context on both the global and local levels would be useful in delimiting inferences, disambiguating deictic expressions, and solving the problem of indeterminacy.

Thus, the distinction of local vs. global can be found elsewhere in the literature though not with the same meaning. Bazzanella (1998), Akman and Bazzanella (2003), and Miecznikowski and Bazzanella (2007) refer to local context to denote a specific setting where the participants interact; and use global context for referring to the members of a community, their social norms, culture, beliefs, ideology, etc. In the same way, Mihalcea (2007) uses the same distinction to refer to a different context span within textual excerpts (a pair of words vs. lexical chains), whereas Dash (2008) proposes a continuum of four contexts from local to global: (i) local context (the immediate environment of a word); (ii) sentential context (syntactic-based); (iii) topical context (domain-based); and (iv) global context (extralinguistic reality).

In our view, local contexts are usually limited to the words within the term itself, to a small number of words in the immediate vicinity of a term, or to words connected by syntactic dependencies to the term. According to Agirre and Stevenson (2007, p. 225), the data that can be derived from local contexts are the following: part of speech, morphology, collocations, subcategorization, frequency of senses, syntagmatic and paradigmatic word association, selectional preferences, semantic roles, domain, topical word association, and pragmatics. Evidently, these categories are not watertight containers since there is a great deal of overlap between information types but they are all valuable data categories to be included in a TKB.

In contrast, global contexts can encompass the whole text or go beyond the text: to the communicative situation (i.e., formal vs. informal); to the conceptual networks reflected in it; to the culture in which the text is interpreted, etc. This means that global contexts refer to items that are often quite a distance from the term or even outside of the text altogether though within the specialized domain.

Both local and global contexts can be subdivided, based on whether they are mainly syntactic, semantic, or pragmatic. In our opinion, it is extremely difficult to trace a clear boundary line between syntax, semantics, and pragmatics because there is a significant degree of overlap. In fact, in Cognitive Linguistics, the distinction between semantics and pragmatics is even rejected. For example, at the local level, the use of term variants *drainage basin* or *catchment area* instead of *river basin* or *watershed* (all expressing the same concept) has pragmatic significance since it signals that the text sender has expert knowledge and is British or Australian instead of American. However, the choice of *drainage basin* also has a semantic dimension since *drainage* foregrounds water movement and accumulation which are the processes that occur in this area whereas *river basin* only foregrounds the location of the basin without any reference to water flow.

At the same time, the term also possesses a syntactic dimension. *Drainage* modifies *basin*, the head of the multi-word term. The implicit relation between modifier and head can be expressed by the preposition *for* (basin for drainage) since the basin is where drainage occurs. However, the structure of the term can be unpackaged to *basin where water drains in and then drains out*. It is thus the result of meaning compression given the fact that *drainage* encodes both the incoming and outgoing flow of water.

This interaction reflects the fuzzy boundaries between syntax, semantics, and pragmatics in general and specialized language. As Dash (2008, p. 29) points out, each context is interlinked with the other in an invisible thread of interdependency. This fuzzy three-level approach to context goes hand in hand with the micro-theories proposed by FBT, which are related to the information encoded in term entries, the relations between specialized knowledge units and the concepts that they designate (Faber, 2015, p. 15).

### Local Contexts

Local contexts are generally regarded as spans of +5 items before and after the term occurrence. They are important in the design stage of a TKB for a wide variety of reasons, which include (but are not limited to): (i) term disambiguation; (ii) meaning definition formulation; (iii) specification of linguistic usage; (iv) conceptual modeling; and (v) term extraction. Thus, local contexts can be used either by resource creators in order to develop terminological by-products (i.e., definitions, conceptual networks, usage examples, etc.); or by the users themselves (i.e., obtaining direct access to the corpus).

In Corpus Linguistics, a recurring local context is known as a *collocation*. However, *collocation* is a rather vague term that does not cover the same range of linguistic phenomena for all linguists (Mollin, 2009, p. 176). The definition of a collocation, its length, the neighborhood of possible collocates and their strengths of occurrence (*inter alia*) are all part of the analysis of specialized language texts and the terms that they contain. Needless to say, the representation of this type of information should be an important element in the design of data fields in terminological entries. However, there are different ways to approach collocational information: they can be seen as a combination of grammatical elements, as the codification of semantic relations, or as pointers to pragmatic information.

Therefore, such local contexts can be syntactically parameterized based on syntagmatic patterns and/or semantically mapped in terms of the interaction, foregrounding, or specification of the definitional features of the concepts activated in them. As shown in Section “Local Pragmatic Contexts,” certain types of pragmatic information are also reflected in local contexts. This occurs, for instance, when term variants indicate changes in the knowledge area, specialization level, geographic region, cultural community, and/or historical period.

#### Local Syntactic Contexts

Local syntactic contexts are those that reflect the recurrent structural patterns in which the term participates. Terms have a combinatorial value and distinctive syntactic projections. However, a term’s position in a subject or direct object slot or as the head of a prepositional phrase is often not very informative since the fact that a term has a certain grammatical function in a sentence is not always relevant to its meaning. Nor is the analysis of a multi-word terminological unit as a mere combination of grammatical categories much more helpful unless this pattern is linked in some way to its underlying semantics. It is more productive to take a semantic view of syntax and to analyze syntactic contexts as the linguistic codifications of predicate-argument structure.

In this regard, each predicate can be said to have an argument structure or valence, specifying the number of arguments that it can take. The concept of valence was first proposed by Tesnière (1959) and now plays a crucial role in the majority of today’s linguistic theories. Generally speaking, valence is regarded as the ability of certain lexical units (e.g., verbs) to open slots which are filled by other lexical units. Valence can be envisaged syntactically, semantically or as a combination of the two. Again, this is proof of the fuzzy interaction of context types and parameters.

A predicate’s valence depends on its meaning since its arguments are essentially the participants which are minimally required for the activity or state described. Such representations should thus include the decomposition of the predicate and the specification of the semantic characteristics of the arguments (Faber and Mairal Usón, 1999).

Despite the fact that verbs have never been a primary focus in Terminology, approaches to syntax in Terminology can benefit greatly from linguistically sensitive theories of lexical structure that focus on verbs and on how their meaning relates to syntactic forms within a sentence. One reason for this is that verbs play an important role in specialized discourse because their position in a lexical domain and degree of semantic specificity is in direct relation to the number and type of arguments that they can combine with (Faber and Mairal Usón, 1999). In specialized texts, these arguments are terms or specialized knowledge units, whose semantic characteristics constrain the polysemy of the verb and even model its meaning. In this sense, one can say that the meaning of general language verbs can be significantly modified or even transformed by their context of activation. When general language verbs appear in domain-specific texts, they become specialized because their arguments constrain their meaning (L’Homme, 2003). At the same time, the presence of a particular verb also constrains the type of argument slots that specialized terms may fill.

For example, *dissipate* is a polysemic general language verb, which is often found in scientific discourse. When it is used transitively in the sense of one entity dissipating another entity, it has two arguments. The first argument has the semantic role of *agent* and the second has the role of *theme*. In this regard, the argument structure of *dissipate* is fairly straightforward since X (agent) causes Y (a theme undergoing the action) to be dissipated:

(1) Dissipate (x)_agent_ (y)_theme_

According to the *Merriam–Webster Dictionary*, this transitive use of *dissipate* has one of the following four senses: (i) to break up and drive off (as a crowd); (ii) to cause to spread thin or scatter and gradually vanish; (iii) to lose (as heat or electricity) irrecoverably; (iv) to spend or use up wastefully or foolishly. Contextual data extracted from the enTenTen12 general English corpus in *Sketch Engine* show that *dissipate* is often used unaccusatively. In other words, the first argument is not made explicit. The most frequent meanings of *dissipate* in general language are ii (2) and iv (3):

(2) To cause to spread thin or scatter and gradually vanish
  (i) Temperature (e.g., *warmth, heat*). [When you exercise on land, sweat evaporates, and cools your skin to **dissipate** *heat.*]
  (ii) Meteorological phenomena (e.g., *storm, fog, mist*). [By afternoon, however, the air traffic from the city had become normal again when the *fog* **dissipated** almost completely.]
  (iii) Visual/olfactory perception (e.g., *mirage, smell*). [I hope most of the *smell* **dissipates** by the time that I ride my bike this afternoon.]
  (iv) Emotions/feelings (e.g., *fear, anxiety*). [With the knowledge, the *anger* **dissipated** as quickly as it had come.]
(3) *To spend or use up wastefully or foolishly*.
  (i) Valuable possessions (e.g., *wealth, resources*). [The entrepreneurs have become wealthy while showing how extravagance and luxuries **dissipate** *wealth.*]

As can be seen in the case of sense (2), the dissipated entity is most frequently related to temperature, weather, sensory perception, and emotions, whereas in sense (3), it is generally wealth or financial resources.

However, in specialized contexts, the meaning of *dissipate* does not really correspond to any of these possibilities. The reason for this is the semantic content of domain-specific arguments, which interact with the base meaning of *dissipate* and model it to create a new sense that is apt for scientific contexts. In the EcoLexicon corpus (subdomain Coastal Engineering), the statistically significant collocates of *dissipate* in the theme slot are the following: (i) *energy* (e.g., *flux, gradient, power*); (ii) *cyclone* or a storm-related term (*tide, wave, wind*, etc.), which can also be regarded as a type of energy. More specifically, energy appears as the generalization of heat whereas cyclone is a specification of energy. **Table 1** shows examples of specialized contexts for *dissipate.*

**Table 1:** Contexts of *dissipate* in specialized texts.

Concordances	Pred-arg structure
(4) Energy (*energy*)	
The wave *energy* has been **dissipated** by wave *breaking* and *bottom friction*.	Dissipate (wave breaking and bottom friction)_agent_ (energy)_theme_
Part of the *energy* is **dissipated** by *breaking processes*.	Dissipate (breaking processes)_agent_ (energy)_theme_
X is the fraction of *energy* **dissipated** by the *falling* sand grains.	Dissipate (falling sand grains)_agent_ (energy)_theme_
Part of the wave *energy* is **dissipated** by the *uprushing* water body.	Dissipate (uprushing water body)_agent_ (energy)_theme_
(5) Meteorological phenomenon (e.g., *cyclone, hurricane, tornado*)	
Only if the *tropical cyclone* **dissipates** with just a tropical disturbance remaining with the OAR give the system a new name.	Dissipate (ø)_agent_ (tropical cyclone) _theme_
*Hurricanes* **dissipate** when their energy supply is substantially reduced.	Dissipate (ø)_agent_ (hurricane) _theme_
Even though the *tornado* is **dissipating**, the tornado is still capable of causing damage.	Dissipate (ø)_agent_ (tornado) _theme_


As can be observed, the arguments of *dissipate* are all NPs that belong to the same semantic categories and combine in similar patterns. In (4) in **Table 1**, the first argument is a process involving some type of friction and the second argument is energy. In (5) in **Table 1**, the storm entity appears unaccusatively without explicitly referring to the reasons for energy loss, which reflects the fact that the target audience is already aware that reasons for storm dissipation include colder sea surface temperatures, shearing winds, sinking air, moving over land, depending on their type, location, and intensity.

In both cases, the interaction of the semantic characteristics of both the dissipated entity (*energy*) and dissipating process (*friction, breaking, falling, uprushing*) clearly point to a new (specialized) sense of *dissipate*, which responds to the Laws of Thermodynamics:

(6) To cause (energy) to be lost through its conversion to heat.

The definition of *dissipate* in (4) fits the domain of Coastal Engineering. The energy produced by wave movement is *dissipated* (lost), typically from friction or turbulence when the waves are near the shore and come into contact with the sea bottom. Of course, the energy is not actually lost but rather is transformed into heat, which raises the temperature of the system. The conversion to heat, though explicit in the definition, is not lexicalized in contexts since it is part of the shared knowledge in the domain. This is one example of how verbs within domain-specific contexts become transformed when they are used in specialized texts since the terms that fill the slots in their argument structure contextualize, modify, and/or restrict their meaning. Moreover, since arguments are specialized terms and verbs are relational constructs, the analysis of argument structure can lead to the construction of semantic networks or frames, which again reflects the fuzzy boundaries between syntax and semantics. For instance, all arguments in the second specialized sense of *dissipate* are cohyponyms (*tropical cyclone*, *hurricane*, *tornado*).

Another way of viewing a local syntactic context is as a *colligation*, initially defined as the co-occurrence of grammatical categories (Firth, 1968, p. 181) or the grammatical company that a word keeps. In multi-word expressions (MWEs), the relations between words and their grammatical categories cover a wide spectrum. In most cases, the words are linked by both grammatical and lexical relations. In fact, it is difficult if not impossible to determine which relation is stronger in each case.

According to Hoey (2005, p. 43), the basic idea of colligation is that in the same way that a lexical item can be primed to occur with another lexical item, it can also be primed to occur in or with a particular grammatical function. Colligation is concerned with the typical grammatical patterning of words (or word classes). As such, collocation and colligation are not totally separate concepts, but together create a network of meaning. Distinguishing collocations (co-occurrences of words) from colligations (co-occurrence of word forms with grammatical phenomena; Gries and Divjak, 2009) is not always a simple task. There is no clear boundary between various types of word combinations inasmuch as they can be simultaneously a collocation and a colligation.

This highlights the interdependence of syntax and lexis. For example, whereas V + *out* + NP is a colligation, *spew* (V) + *out* + *air pollution* (NP) is a collocation which exemplifies the colligation. However, the meaning of *colligation* has since expanded to include the specification of semantic preference or semantic prosody. Accordingly, it can now refer to the co-occurrence of lexis and grammatical categories.

Semantic preference is the “relation between a lemma or word-form and a set of semantically related words” (Stubbs, 2002, p. 65). Semantic prosody (Louw, 1993) captures the fact that some elements attract lexical items designating negative things, features, actions, etc., whereas others show a characteristic co-occurrence with positive elements. Together these notions expand into the notion of semantic colligation: the mutual attraction holding between a grammatical construction and a semantic category (Gabrielatos, 2007).

For example, when something is projected (*spewed, pumped*, etc.) from a container, the ejected entity (*air pollution, CO_2_*) is frequently undesirable. Moreover, this semantic preference is confirmed by corpus data showing that when *pollution* is the theme argument, it tends to consistently combine with verbs belonging to two semantic domains:

(i) Change: to cause to become less (*reduce, decrease, abate, minimize, mitigate, lessen, cut*).
(ii) Movement: to move out of/from a certain place (*spew, emanate* with the focus either on the endpoint of the trajectory (*pollution spewed into the air/environment*) or its starting point (*pollution spewed from the Chinese mine/coal-fired power plant/plane*).

When *pollution* or one of its components is the agent argument, it also tends to combine with verbs of change, but primarily with those predicates belonging to the subdomain *to cause something to become worse* (*contaminate/foul/degrade*, etc.).

(7) Still more fluorides from such pollution ***contaminate*** the animals and plants we use as food.
(8) The measure should help rescue the Chesapeake Bay from the nutrient pollution ***fouling*** its water.
(8) Changes in the flow of the river and pollution have very severely ***degraded*** the ecosystem.

Optionally, it is also activated with verbs of causative existence in the subdomain of *to cause something bad to happen.* More specifically, *threaten* means *to cause something or someone to be vulnerable or at risk.*

(10) The oceans are now so ***threatened*** by pollution and exploitation that many shorelines will soon be totally denuded of marine life.

The predisposition to appear in certain syntactic structures and combine with predicates from specific semantic subdomains is directly related to the semantic load of *pollution*, a dot object according to the (Generative Lexicon) (Pustejovksy, 1995), which can be regarded either as a process or the result of a process.

Both colligations and predicate argument structures reflect the fuzzy boundary between syntax and semantics. The fact that words that occur together tend to be semantically similar explains that local syntactic contexts could also be used for semantic clustering. Popularized by Firth (1957) in his famous line “a word is characterized by the company it keeps,” this approach has been implemented in the distributional hypothesis (Harris, 1985) and the strong contextual hypothesis (Miller and Charles, 1991). Furthermore, within the scope of a sentence, word sense disambiguation is usually determined by a combination of two factors: (1) the syntactic frame into which the word is embedded, and (2) the semantics of the words with which it forms syntactic dependencies (Rumshisky, 2008, p. 217).

#### Local Semantic Contexts

Local semantic contexts can either refer to semantic relations between the constituents of the specialized knowledge unit (term-internal semantic context) or to semantic relations between different specialized knowledge units in the text (term-external semantic context). In the first case, the scope of the context is the multi-word term itself, whose interpretation is based on the meanings as well as the dependency relations between the head and the modifiers from which a semantic relation can be inferred. In the second case, the context is the linguistic codification of a triplet: two specialized knowledge units linked by a phrase that explicitly marks a semantic relation.

*Term-internal semantic contexts* are exemplified by MWEs and constitute a large portion of the lexicon of any natural language. It is estimated that the number of MWEs in the lexicon of a native speaker is the same as the number of single words (Jackendoff, 1997), and these ratios are probably even higher in the case of domain-specific language, in which the specialized vocabulary and terminology are composed mostly of multiword expressions. According to Erman and Warren (2000, p. 29), the fact that half of spoken and written language comes in pre-constructed multiword combinations makes it impossible to consider them as marginal phenomena. In fact, these specialized MWEs are rapidly increasing because of the continuous addition of new terms that designate new concepts. This makes it virtually impossible to store all of them in a dictionary.

Since the meaning of multi-word terms is often a specialization of the meaning of its head, in many cases, term structure can be used as a way to automatically extract information for the specification of conceptual hierarchies, one of the main components of TKBs. In morphologically poor languages, such as English, they can take the form of sequences or stacks of nouns of varying length: (i) two constituents (*capillary wave*); (ii) three constituents (*long-wavelength surface wave*); (iii) four constituents (*ocean surface gravity wave*); and (iv) five constituents (*surface gravity wave elevation spectra*). It is for the addressee to unpack their meaning and determine the implicit relationship between the constituents. One way of doing this is to understand such compounds in terms of left or right branching dependency relations:

(11) [capillary (wave)]
(12) [long-wavelength (surface wave)]
(13) [ocean (surface gravity wave)]
(14) [((surface gravity wave) elevation) spectra]

Evidently, the more numerous the terms in the stack, the more difficult it is for a computer to automatically establish dependencies. The most complicated cases are (13) and (14). For example, in (13), it is necessary to know that the term must be interpreted as a right-branching compound, since the term does not refer to an ocean surface. Instead, *surface gravity wave* designates an important type of wave (which is a synonym of *surface wave)*, which propagates in the ocean. In contrast, (14) is a left-branching compound, which is gradually generated from *surface gravity wave* by adding the subsequent heads of *elevation* and then *spectra.* The analysis of these dependencies is often based on term entrenchment and extremely difficult for a computer to perform automatically unless it has been previously trained to do so.

Another way of understanding term creation is to think of the general concept as an entity with an underspecified meaning. Its nature is what predetermines the potential specification of its meaning that will lead to the generation of hyponyms.

For example, *wave* designates an oscillation propagating through a medium. As such, it has a series of basic defining attributes, such as height, wavelength, steepness, period, speed, and frequency. This can be regarded as a kind of frame or type of implicit context that opens up slots. The specification of one or more of these attributes that modify the head (*wave)* creates different wave types (*long-period wave, high-frequency wave*, etc.).

However, these defining attributes are not the only source of new terms. When a wave is regarded as a process, this creates a more abstract context or frame that opens the door to other possible subtypes. A wave can thus be regarded as caused by an agent (*wind wave*), affected by a force (*gravity wave*), moving in a certain way, (*plunging breaking waves*), taking place at a certain location (*surface wave*), and occurring in a certain medium (*water wave).* However, in some cases, both scenarios or contexts can combine to produce terms such as *long-wavelength surface gravity wave*. This term has the following meaning relations:

(15) Long-wavelength surface gravity wave

**Head**: wave

**Located_at**: surface

**Affected_by**: gravity

**Length_of** : long-wavelength

*A long-wavelength surface gravity wave* can thus be regarded as an oscillation (*wave*) with a long wavelength (*long-wavelength*) on the air–sea interface (*surface*) affected by a restoring force (*gravity*).

Evidently accessing the meaning of such a combination is not a trivial process since it activates a whole specialized frame that requires previous knowledge. In this sense, Maguire et al. (2010, pp. 49–50) cite the concept specialization model (Murphy, 1988) and dual process theory (Wisniewski, 1997), which propose a two-stage interpretation process. The first stage involves a slot-filling mechanism where the modifier is inserted into a slot in the head-noun schema to form an interpretation. The type of noun modifier is directly related to the basic meaning of the head.

For instance, in *N* + *N* compounds in which *energy* is the headword, the slot activated is usually agent (e.g., *wave energy, wind energy, heat energy*, etc.), which highlights the source or natural force producing the energy. In contrast, in sediment compounds, in most cases, the headword opens a <location> slot (e.g., *intertidal zone sediment, streambed sediment, aquifer sediment*, etc.) since sediment is solid fragmented material that is transported and deposited by water or wind at a certain location. Alternatively, there is also a set of sediment terms in which a <made-of> slot is opened up (*lithogenous sediment, biogenous sediment, hydrogenous sediment, cosmogenous sediment*). These A + N compounds foreground the “fragmented material” part of the definition.

Consequently, this indicates that the membership of the head noun in a small number of broad semantic categories reveals consistent patterns in modifier and head use, and that the semantic categories are not randomly paired.

*Term-external semantic contexts* take the form of KWIC concordances or knowledge-rich contexts that provide information about a concept’s attributes or the relations that it forms with other concepts. They contain Knowledge Patterns (KPs; Barrière, 2004), which are lexico-syntactic patterns that indicate a semantic relationship, and at least two specialized knowledge units. Studies in this tradition include Pearson (1998), Meyer (2001), Barrière (2004), Aussenac-Gilles and Jacques (2006), and Sierra et al. (2008), *inter alia*.

Mitkov (1998, 2002) and Meyer (2001) distinguish between knowledge-rich contexts and knowledge-poor contexts. Knowledge-poor contexts do not include any item of domain knowledge related to the search word. In contrast, knowledge-rich contexts contain at least one item of domain knowledge that is useful for the conceptual analysis of the search word. Such contexts should indicate at least one conceptual characteristic, whether it is an attribute or relation (Meyer, 2001, p. 281).

**FIGURE 1:**
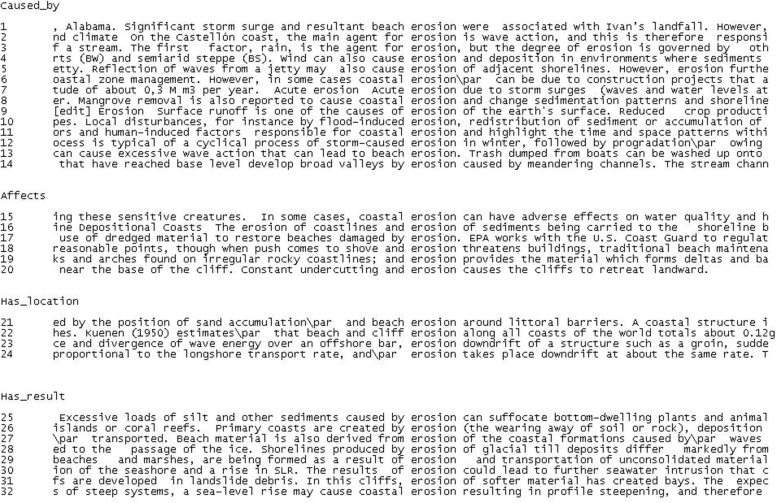
**Non-hierarchical relations associated with EROSION**.

For example, the concordances of *erosion* in **Figure 1** show how different KPs convey different relations with other specialized concepts. The main relations reflected in erosion concordances are *caused_by*, *affects*, *has_location*, and *has_result*, which highlight the procedural nature of the concept and the important role played by non-hierarchical relations in knowledge representations.

In **Figure 1**, erosion is related to various types of agent, such as storm surge (1, 7), wave action (2, 13), rain (3), wind (4), jetty (5), construction projects (6), mangrove removal (8), surface runoff (9), flood (10), human-induced factors (11), storm (12) and meandering channels (14). They can be retrieved thanks to all KPs expressing the relation *caused_by*, such as *resultant* (1), *agent for* (2, 3), *due to* (6, 7), *responsible for* (11), and *lead to* (13). This relation can also be conveyed through compound adjective phrases, such as *flood-induced* (10) or *storm-caused* (12) and any expression containing *cause* as a verb or noun: *one of the causes of* (9), *cause* (4, 5, 8), and *caused by* (14).

Erosion is also linked to the patients it *affects*, such as water quality (15), sediments (16), coastlines (16), beaches (17), buildings (18), deltas (19), and cliffs (20). However, the affected entities, or patients, are often equivalent to locations (e.g., if erosion *affects* beaches it actually *takes place at* the beach). The difference lies in the KP linking the propositions. The *affects* relation is often reflected by the preposition *of* (10) or by verbs such as *threatens* (18), *damaged by* (17) or *provides* (19). In contrast, the *has_location* relation is conveyed through directional prepositions (*around*, 21; *along*, 22; *downdrift*, 23) or spatial expressions, such as *takes place* (24). In this way, erosion is linked to the following locations: littoral barriers (21), coasts (22), and structures (23). *Result* is an essential dimension in the description of any process since it is not only initiated by an agent affecting a patient in a particular location, but also has certain effects, namely, the creation of a new entity (sediments, 25; primary coasts, 26; beach material, 27; shorelines, 28; marshes, 29; bays, 31) or the beginning of another process (seawater intrusion, 31; profile steepening, 32).

**FIGURE 2:**
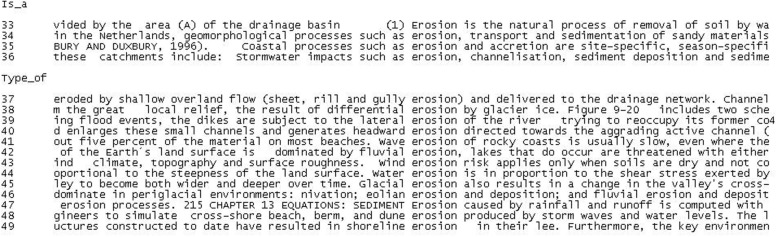
**Hierarchical relations associated with EROSION**.

As can be seen, all these related concepts are quite heterogeneous. They belong to different paradigms in terms of category membership and/or hierarchical range. For instance, some of the agents of erosion are natural (wind, wave action) or artificial (jetty, mangrove removal) and others are general concepts (storm) or very specific ones (meandering channel). This explains why knowledge extraction must still be performed manually or semi-automatically and how local semantic contexts can be conceptually valuable. Nevertheless, it also illustrates one of the major problems in knowledge representation: *multidimensionality*. *Multidimensionality* has been defined by many authors (Bowker, 1997; Kageura, 1997; Wright, 1997; Rogers, 2004) as the phenomenon in which certain concepts can be classified according to different points of view or conceptual facets. Evidently, multidimensionality has important consequences regarding how domains are categorized and modeled (León-Araúz et al., 2013). This is better exemplified in the concordances shown in **Figure 2** since multidimensionality is most often codified in the *is_a* relation.

In the scientific discourse community, concepts are not always described in the same way because they depend on perspective and subject-fields. For instance, *erosion* is described as a natural process of removal (33), a geomorphological process (34), a coastal process (35), or a stormwater impact (36). The first two cases can be regarded as conventional ontological hyperonyms. The choice of one or the other depends on the upper-level structure of the representational system, its level of abstraction and the support for context. However, the other two cases (i.e., coastal process and stormwater impact) cause the concept to be framed in more concrete subject-fields and referential settings.

The multidimensional nature of erosion is also clearly shown in subtypes, which are codified in term-internal semantic contexts. Erosion can thus be classified according to the dimensions of *result* (sheet, rill, gully, 37; differential erosion, 38), *direction* (lateral, 39; headward erosion, 49), *agent* (wave, 41; fluvial, 42; wind, 43, 46; water, 44; glacial erosion; 45), and *patient* (sediment, 47; dune, 48; shoreline erosion, 49).

These dimensions are contexts that need to be specified in the TKB in order to delimit information retrieval and make it more relevant. They can be represented as part of a definitional template (all cohyponyms being defined according to the same dimensions). Alternatively, they can be codified as a specification of the subsumption relation (fluvial erosion *is_a (agent)* erosion), or simply as a concordance or knowledge-rich context.

**FIGURE 3:**
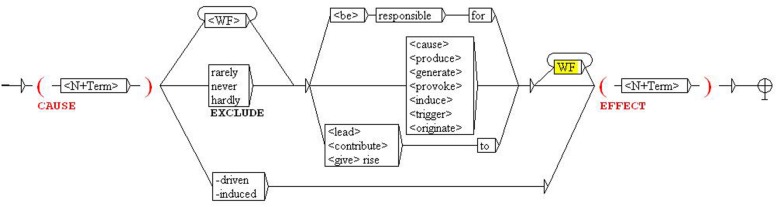
**Local grammar for causal relation extraction**.

In order to retrieve new related term pairs, KPs can be collected and systematized in the form of local grammars. For instance, **Figure 3** shows part of the formalization of the causal relation, which is based on causative verbs in any of their inflected forms (*cause*, *produce*, *generate*, *trigger*), morphological particles (-*driven*, -*induced*) and other literal causative expressions (*responsible for*), as exemplified in the concordances of erosion (**Figure 1**). When this grammar is applied to the corpus, it identifies structures such as “tsunamis, usually caused by large earthquakes” or “rain produced severe flooding,” from which we can derive the conceptual propositions, or triplets, TSUNAMI *causes* EARTHQUAKE and RAIN *causes* FLOODING.

Once again, syntactic and semantic local contexts are not discrete variables. However, this approach must also be contrasted with global semantic and pragmatic contexts, since conceptual knowledge as reflected in the text is not always reliable. This means that texts do not reflect perfectly designed conceptual networks. For instance, in hyponymic term-external semantic contexts (*x such as y, y and other x, x is a type of y*, etc.), authors do not always choose the direct parent of a concept. Many times, they will use a grandparent (WORK> GROIN instead of COASTAL STRUCTURE> GROIN) or will even create an *ad hoc* category (OBSTACLE TO FLOW> GROIN instead of COASTAL STRUCTURE> GROIN; León-Araúz and Reimerink, 2016).

Furthermore, the existence of multiple hyperonyms can indicate two types of multidimensionality: intracategorial and inter-categorial multidimensionality. In intracategorial multidimensionality, hyperonyms point to the same concept but highlight different dimensions or different levels of granularity. However, in intercategorial multidimensionality, hyperonyms point to a paradigm change, which makes the different facets incompatible.

One example is FOREST, which is found in local contexts as a type of ecosystem or as a type of renewable resource. This means that the concept is viewed as a type of one hyperonym to the exclusion of the others. This evidently affects the way in which the concept relates to other concepts. For instance, when a forest is viewed as a renewable resource, it is more closely related to concepts such as solar energy and biofuel, whereas when viewed as an ecosystem, wetlands and lakes are its closest concepts. This necessarily has an impact on knowledge and context modeling. Contrasting these results with a global approach (see section “Global Contexts”) and analyzing lexical cohesion in the whole text where these structures occur can result in a reliable reconstruction of a text-driven conceptual system.

#### Local Pragmatic Contexts

Local pragmatic contexts basically refer to parameters of terminological variation and culturemes. Although in Terminology, the initial goal was to have one linguistic designation for each concept for greater precision, it soon became obvious that in descriptive terminology work, this is not always the case. This occurs more frequently in standardization settings (e.g., institutional, legal, technical, etc.) where the objective is to harmonize terminologies for the sake of efficient unambiguous communication. However, in the same way as for general concepts, the same specialized concept can often have many linguistic designations depending on the context. Alternatively, the same linguistic designation can also refer to various concepts.

As in general language, it is possible to establish reasons for terminological variation based on user-based parameters of geographic, temporal or social variation or usage-based parameters of field, tenor, and channel (Gregory and Carroll, 1978). Nevertheless, these basic parameters only provide a very partial representation of a very complex situation, since there are other reasons for terminological variation that are often considerably more difficult to represent.

Freixa (2006, p. 52), for example, classifies the causes for terminological variation in the following categories: (1) dialectal, based on the origin of the authors; (2) functional, based on communicative registers; (3) discursive, based on the style of the authors; (4) interlinguistic, based on the contact between languages; and (5) cognitive, based on different conceptualizations. These are all pointers to different types of extra-textual contexts, which mainly stem from the author’s identity, location, language, and way of thinking. According to Freixa (2002), cognitive term variants are not only formally different, but also semantically diverse, as they give a particular vision of the concept. They are thus the natural reflection of multidimensionality (Fernández-Silva et al., 2011). Very often, the choice of one term instead of another stems from different perspectives of reality. Nevertheless, there are certain types of variation that do not fall into any of these categories, such as morphological variants, orthographic variants, ellipted variants, abbreviations, graphical variation, variation by permutation, etc. (Bowker and Hawkins, 2006, p. 81). Their use in texts often seems to be random without responding to any pattern or regularity. Although initially, the existence of such variants may not seem to be a problem, reality is somewhat different. Since term variants are rarely interchangeable, it is not a question of merely adding more terms to the TKB. What is needed is more information in term entries so that users can know which term to select. In terminological resources, users are often confronted with a vast array of variants with no indication of how term variation arises or how their use may be constrained.

In fact, variants often have a communicative and/or cognitive motivation. Therefore, the use of one term or another may affect the semantics of a concept or the communicative situation in which the concept is activated. Based on this distinction, our experience in EcoLexicon and other foundational work on term variation, we propose that term entries should include the following extended classification of pragmatic markers. When building a multilingual TKB, these markers can also enhance interlingual correspondences, because users will be able to make a cognitively sound choice. Otherwise, translators may actually over-standardize, creating consistency in places where the use of variants was deliberate and well-reasoned (Bowker and Hawkins, 2006, p. 80).

(A) *Orthographic variants* (with no geographic origin, e.g., *aesthetics*, *esthetics*), which do not affect semantics or the communicative situation.
(B) Diatopic variants
  (i) *Orthographic variants* (e.g., *groyne*, *groin*), which do not affect semantics.
  (ii) *Dialectal variants* (e.g., *gasoline*, *petrol*), which may affect semantics if culture-bound factors highlight or suppress any of the semantic features.
  (iii) *Culture-specific variants* (e.g., *dry lake*, and *sabkha*), which affect both semantics and the communicative situation. When referring to a particular entity that, in a specific culture, adds more specific features, culture-specific terms are used as variants of the closer entity in the target culture.
  (iv) *Calques*, which may affect semantics and the communicative situation and are the result of an interlinguistic borrowing for different reasons, such as the impact of a certain language on a specialized domain or the inexistence of the entity or term in a particular language.
(C) *Short form variants*, which do not affect semantics but only the communicative situation.
  (i) Abbreviation
  (ii) *Acronym* (e.g., *laser*, *Light Amplification by Stimulated Emission of Radiation*).
(D) Diaphasic variants
  (i) *Scientific variants*, which do not affect semantics but only the communicative situation.
    (a) *Scientific names* (e.g., *Dracaena draco*, *drago*), which refer to specialized nomenclatures and are especially useful in botany, zoology, chemistry, etc.
    (b) *Expert neutral variants* (e.g., *Ocellaris clownfish*, *Amphiprion ocellaris*), which would be the default term choice in a specialized scenario.
    (c) *Jargon*. Sometimes experts have their own informal way to refer to specialized concepts (e.g., in medicine, *lap-appy* would correspond to *laparoscopic appendectomy*, but no lay user would use this term).
    (d) *Formulas* (e.g., *H_2_O*, *water*; *CaCO_3_*, *pearl*), which do not affect semantics but only the communicative situation. *H_2_O* and *water* do not change in meaning but the formula imposes certain constraints on text senders and receivers.
    (e) *Symbols* (e.g., *€*, *euro*).
  (ii) *Informal variants*, which do not necessarily affect semantics but especially the communicative situation.
    (a) *Lay user variants* (e.g., *Dragon tree, drago*), which would be the default term choice in non-specialized scenarios.
    (b) *Colloquial variant* (e.g., *hydraulic fracturing*, *fracking*).
    (c) *Generic variants* (e.g., *sea*, *ocean*; *weathering*, *erosion*). Very informal variants can activate terms pointing to different levels of conceptual granularity and thus affecting semantics.
  (iii) *Domain-specific variants* (e.g., *mud*, *sludge*), which may affect semantics and/or the communicative situation when term preferences change across specialized domains.
(E) *Dimensional variants* (e.g., *Gutenberg’s discontinuity*, *core-mantle boundary*), which are usually multi-word terms and affect semantics, since they convey different dimensions of the same concept (*Gutenberg’s discontinuity* activates the person who first named it and *core-mantle boundary* the two areas that it separates).
(F) *Metonymic variants* (e.g., *water*, *sea*), which affect semantics because the metonymic variant designates the parts or material that the concept is made of.
(G) *Diachronic variants*.
(H) *Non-recommended variants* (e.g., in medicine, *mental retardation* now has negative connotations and has been substituted by *intellectual disability*).
(I) *Morpho-syntactic variants* (e.g., *wave action*, *the action of the waves*), which do not affect semantics but depend on collocates, term selection preferences, and the communicative situation.

The nature and scope of these variants are very diverse. Furthermore terms can activate more than one type of variant, which might make term choice more difficult. For example, *H_2_O* and/or *water* are domain-based variants since the first is more frequently used in chemistry and water treatment domains than in hydrology or geology. However, their use also depends on the communicative situation and the knowledge level of the speaker and receiver. In the same way, *lap-appy* could be classified as jargon as well as a short form. On the other hand, the same type of variant can also be expressed by more than one term. Diaphasic variants, in particular, form a continuum ranging from more formal to informal (e.g., *thermal low pressure system*, *thermal low*, *thermal trough*, and *heat low*). The same happens within expert variants, which can be graded on a scale of frequency or acceptance. For instance, coastal defense and coastal management are both expert variants, but, coastal management is the preferred term.

Moreover, in those cases where the same concept can be designated by different dialectal variants stemming from the geographic origin of the writer, this can also mean that, conversely, the same term can be used to designate different entities in different cultures. For instance, *pier* (a structure built on posts extending from land out over water, used as a landing place for ships, an entertainment area, or a promenade area) is often designated as *jetty* in the Great Lakes. In contrast, a jetty is most often a structure designed to prevent the shoaling of a channel and is not considered a recreational area. However, in British English, *jetty* is the synonym of a *wharf*, whereas, in American English, *pier* may also be a synonym of *dock*. Nonetheless, in British English a *dock* is the area of water used for loading or unloading cargo in a harbor, which in American English is called a *port*.

Geographical variation in this category domain can often be conceptually motivated and mainly based on the dimensions of location and function. For instance, a dike may be called a *levee* when it is located on a river, whereas a breakwater may be called a *mole* when it is covered by a roadway. On the contrary, when a breakwater serves as a pier, it is called a *quay* in British English and a *wharf* in American English. Needless to say, when the knowledge base includes a conceptual representation or ontology, important design decisions must be taken. A base concept must be chosen (e.g., PIER) and be specified to accommodate the references of these variants (e.g., WORKING_PIER, PLEASURE_PIER, FISHING_PIER, etc.).

Local pragmatic contexts are thus reflected in terms and multiword expressions that are pointers to larger (global) situational, linguistic, and cultural contexts. Therefore, local and global pragmatic contexts constrain each other. Local contexts point to global contexts by constraining all possible situations (i.e., geographic, communicative, cognitive), and global contexts drive the choice of one variant over the rest.

Consequently, term variation should not be regarded as a linguistic phenomenon isolated from conceptual and cultural representations since it is one of the manifestations of the dynamicity of categorization and expression of specialized knowledge (Fernández-Silva et al., 2014).

### Global Contexts

Contexts can also be global with a wider scope. The scope of such contexts can be a whole document, a communicative situation (e.g., formal vs. informal), a subject domain (e.g., Geology, Meteorology, etc.), or an entire language-culture.

Global contexts affect the underlying design of the data fields of a TKB since they are too large to be included in a term entry unless it is in the form of syntactic, semantic, or pragmatic markers, which would be more suitable for local contexts. They can also be analyzed with a view to tagging and classifying corpora in macro- and micro-structural terms.

The macro- and micro-structure of a text, or even a set of texts where intertextuality plays a role in understanding a specialized domain, provide a larger context to be analyzed with regards to grammatical and lexical cohesion (syntactic and semantic context). When global contexts are extra-textual, they are global pragmatic contexts characterized by different combinations of authorship, readership, function, domain, culture, etc.

#### Global Syntactic Contexts

When the document is used as the context, global syntactic contexts consist of the means of grammatical cohesion that tie the text together. These include endophoric reference (anaphora, cataphora), substitution, and ellipsis, as well as other grammatical cohesive markers that connect the different sentences of a text in a logical manner, such as, *however*, *on the other hand*, *consequently*, etc. Such contexts could presumably also refer to the use of verb tenses throughout a discourse. For example, the typical Introduction, Methodology, Results, and Discussion (IMRAD) format of research articles is reflected in the verb tenses used in each section.

These verb tenses set the scene for the description of a research study and the presentation of results. In English, for example, the introduction is generally in the present tense when the author is describing the cause or (problematic) situation that produced the need for the research and the review of the literature on the topic. However, the past tense is used when referring to how the experiment was carried out. The present tense is again used when the author outlines the sections of the article. This use of verb tenses can differ when the article is written in a different language. For instance, in Spanish, there is a greater tendency to use the present and future tenses as though the research being described were being carried out in the paper itself.

Although it might seem that the use of tenses and syntax in general has little impact on terminology structure and selection, this is not the case. As observed by Gotti (2003), the fact that specialized discourse is characterized by elementary surface structures and relatively simple syntax allow the author a certain license to use complex and long pre-modified MWEs, which leads to a far longer sentence length. This means that the use of more or less complex nominal compounds is in direct relation to the relative simplicity of the syntactic structures in the text.

Grammatical cohesion in scientific discourse is often domain-independent, but still specialized. The same happens with the *transdisciplinary scientific lexicon* (Drouin, 2010), which includes abstract verbs (*to think*, *to consider*), abstract nouns (*idea*, *factor*, *relation*, *hypothesis*, *data*, *approach*), and collocations (*to conduct an analysis*) that refer to the description of scientific activities and reasoning but do not point to domain concepts. Thus, the study of global syntactic contexts can also have important computational applications, such as term extraction and coreference resolution.

#### Global Semantic Contexts

Global semantic contexts are in turn reflected in the lexical cohesion of texts (Halliday and Hasan, 1976; Morris and Hirst, 1991). Lexical cohesion is based on the meaning relations between words in a text. Such relations are paradigmatic and link two words having a common component from the viewpoint of their meaning.

Apart from repetition, lexical cohesion is most frequently achieved by using synonyms and hyperonyms, which requires a certain previous knowledge of the domain. In this sense, the description of local semantic and pragmatic contexts in TKBs ensures lexical cohesion when TKBs are used for text production tasks. In fact, it has been shown that scientific journal articles and popularized accounts of the same research do not employ the same cohesive patterns (Myers, 1991). According to Myers (1991, p. 5), the readers of scientific texts must have previous knowledge of lexical relations to see the implicit cohesion of the text, while readers of popularizations must see the explicitly marked cohesive relations to infer lexical relations, and to link the semantic field of the specialized domain to those of everyday life.

Thus, the analysis of lexical cohesive devices, which is hardly a trivial task, has also been approached from a computational perspective. In distributional approaches, synonyms, hyperonyms, antonyms, etc., are typically calculated by means of context vectors for each word, grouping together words that appear in the same contexts. In Ellman and Tait’s (1998) framework, a single instance of lexical cohesion is a lexical link, whereas a sequence of links is a lexical chain. Such chains can also be formed by relations or bonds between sentences that are related by two or three links (Hoey, 1991).

Lexical chains are identified by using relationships between word senses. Nevertheless, in order to build lexical chains, it is necessary to know word senses and semantic relations between words. A lexical chain for a text contains a subset of the words (word senses) in the text, and are semantically related. Although the length of such chains may cover a larger or smaller portion of the text, in this case, we are referring to those that cover the whole document. Evidently, the number of words and the number of semantic relations between words can be different for each lexical chain. According to Ercan and Cicekli (2007), the coverage and size of a lexical chain can indicate how well the lexical chain represents the semantic content of the text. Lexical chains are evidently meaning-based but can also be derived from collocational frequencies. For example, Phillips (1989, p. 51) states that the collocation between *electric* and *charge* is also linked to the patterns in the text between their collocations (e.g., *charge* collocates with *distribution*, *density*, *point*, and *uniform*; *electric* collocates with *dipole*). Bondi (2010, p. 4) affirms that this network of semantic relations identifies the ‘aboutness’ of a text, and is a marker of text content.

#### Global Pragmatic Contexts

Global pragmatic contexts are the most complex form of context to be systematized and should thus be represented in a TKB, since they involve different interrelated variables.

Pragmatics is at the core of the dynamics of both terms and concepts (León-Araúz and Faber, 2014), since changes in conceptualization and in the lexicon are clearly not independent of each other but interact in a number of unforeseeable ways (Cimiano et al., 2010).

Generally speaking, pragmatics focuses on the effect of context on communicative behavior as well as on how inferences are made by the receiver (Faber, 2012). Crucial pragmatic dimensions in specialized communication contexts include (1) the beliefs and expectations of the text sender; (2) the knowledge shared by the text sender and text receivers; (3) the communicative objectives of the oral or written text stemming from the interaction of the participants; and (4) the factors that cause receivers to interpret the text in a certain way (Faber and San Martín, 2012, p. 178).

Strictly speaking, by its very definition, any type of pragmatic context is global. As previously mentioned, even local pragmatic contexts, as reflected in term variants or culturemes, are markers that point to larger communicative and cultural situations, which have an impact on conceptualizations in a given language-culture. Precisely for that reason, the description of entities is necessarily constrained by contextual variation across communicative situations, cultures, and disciplines, as well as the fuzzy category boundaries that they establish.

For example, texts with a high term density (percentage of specialized knowledge units) are written by experts who wish to transmit knowledge to other experts in the same domain. Texts written for semi-experts or for non-experts have a correspondingly lower term density, although more term variants tend to be employed for the sake of transparency.

In specialized communication, genre and register are important concepts even though their definitions often seem to confusingly run together. However, following Lee (2001, pp. 46–47), we use register to refer to lexical-grammatical and semantic discourse patterns associated with situations, whereas genre is used to refer to the membership of a text in culturally recognizable categories, which may invoke more than one register. As such, genre is a socio-pragmatic phenomenon. According to Unger (2002, p. 2), a socio-pragmatic phenomenon is a set of shared assumptions that governs the communicative behavior of members of this group. It also relates communicative behavior to the structure of cultural institutions.

Hoffmann (1985, 1990) states that the purpose of a text depends on the context in which the text was created. In this sense, a text is both an instrument and a result that comes into being because of the specific productive activity (Hoffmann, 1985, p. 233). Similarly, Roelcke (1999, p. 42) underlines the importance of the specialized text regarded as a whole, and observes that the context of language usage also goes hand in hand with an increasing specialization of scientific and professional fields.

Although a definitive inventory and classification of specialized language genres and registers does not as yet exist, specialized language genres would doubtlessly be linked to specialized knowledge activities and text function within the context of a specialized knowledge field (cf. Hoffmann, 1985). Registers would presumably be subdivided primarily according to levels of formality. These formality levels would be constrained by parameters inherent in the context of specialized communication.

However, in TKBs, communicative context should not only be codified as a local pragmatic marker in term entries, especially when the aim of querying a TKB is multilingual communication. The reason for this is the fact that register-based variants in different languages do not necessarily establish 1:1 correspondences. This means that if a concept is designated by an informal term variant, it should not always be translated by its informal counterpart in another language and vice versa, because pragmatic conventions can also change from culture to culture. For instance, in an English doctor–patient communicative act, doctors tend to use more informal variants than in a similar situation in Spain. Even if a term-pair such as *intestinos* and *intestines* are full equivalents, *bowels* would be more appropriate in an English situation.

Nevertheless, the influence of culture is reflected in specialized domains in much more complex ways than it is in culture-specific terms or register-based differences (Faber and León-Araúz, 2014). They also may affect conceptual structures. For instance, one might think that natural landforms are more or less the same all over the world, but the truth is that there is a great deal of plasticity in how language models the earth and what is considered to be the essence of its features (Burenhult and Levinson, 2008, p. 148). Until recently, it was believed that entities such as mountain and river were candidates for universals (Smith and Mark, 2001). However, research in cognitive ethnophysiography has found that this is not the case. Apart from the problem of establishing interlinguistic correspondences, this also makes it hard to agree on how concepts are classified in the same language.

For example, the diversity of wetlands is an obstacle to arriving at a consensus in regards to their classification. One of the most widely used classifications was created by Cowardin et al. (1979), who divided wetlands into marine, estuarine, riverine, lacustrine, and palustrine environments. Nevertheless, this classification was eventually found to be too restrictive, and a more comprehensive categorization was required. The Ramsar classification system for wetland types (1996) thus proposed new categories to cover all types of wetlands in the world: marine/coastal wetlands, inland wetlands, human-made wetlands. In turn, the Canadian national wetlands working group (1997) established five classes: bog, fen, marsh, swamp, and shallow water.

However, labeling categories in terms of basic level concepts (Rosch, 1978) can be confusing, because they are highly localized. For instance, bogs or fens are usually grouped together and referred to as *mires* in Europe, but not in America. Marshes in Europe are often called *reed swamps*, but swamps in America are not dominated by reeds but rather by trees. *Carr* is the northern European term for the Southeast American *wooden swamp*, which in the United Kingdom is also called *wet woodland*. There are also specific types of wetlands that only predominate in certain geographic areas that are not lexicalized in all cultures, such as the Australian *billabong*, the African *dambo*, or the Canadian *muskeg*. In these cases, the local terms are only borrowed when describing these particular wetlands. Thus, when one of these terms is activated in a text, the location-related category features of the concept are constrained.

**FIGURE 4:**
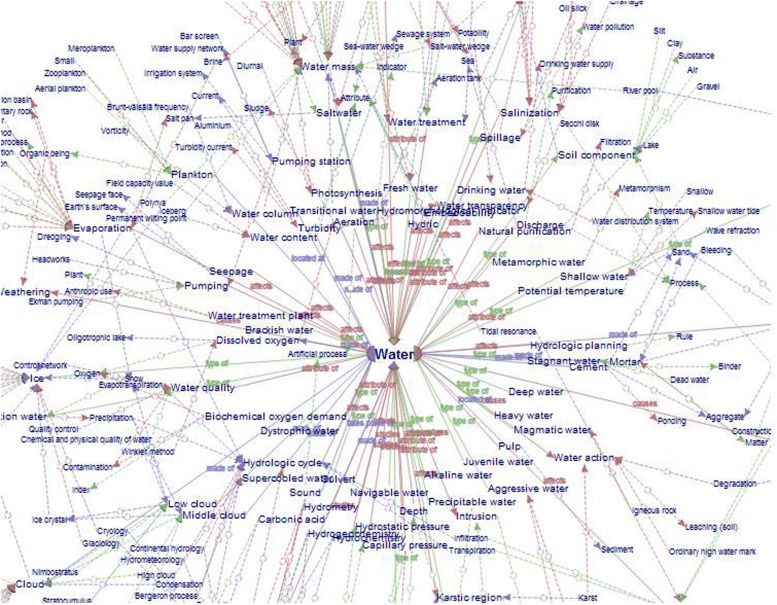
**Information overload in EcoLexicon**.

Multidimensionality is also found in discipline-based contexts. In Terminology, multidimensionality is often regarded as a way of enriching traditional static representations, enhancing knowledge acquisition through different points of view in the same semantic network or conceptual system. However, it can also produce an excessive information load. This is the case of certain general top-level concepts such as water (**Figure 4**), which is a classic example of information overload in EcoLexicon (León-Araúz et al., 2012, 2013; Faber et al., 2014a).

Water certainly holds different relations with a myriad of different concepts. However, EcoLexicon users would not acquire any meaningful knowledge if all dimensions of water were shown in the same network. Moreover, water rarely, if ever, activates those concepts at the same time, since this would evoke completely different and incompatible scenarios (León-Araúz et al., 2013). In this sense, although it is true that concepts cannot be activated in isolation, they can also retain sufficient autonomy so that the activation of one does not necessarily entail the activation of the rest (Langacker, 1987, p. 162). Their activation should thus be domain-dependent.

According to Picht and Draskau (1985, p. 48), multidimensionality depends on the classifier as well as the different knowledge sources that may reflect different criteria when organizing the same domain. In conceptual modeling, facets and contexts can be established according to different criteria. However, in EcoLexicon, a discipline-oriented approach was found to be the most appropriate, since concepts may have different roles and degrees of prominence in the different disciplines that constitute the environmental sciences.

As opposed to formal approaches where concepts are ascribed to particular categories on the basis of a set of necessary and sufficient features, semantic networks in EcoLexicon take the form of a set of conceptual relations that might be highlighted or suppressed, depending on pragmatic factors. We agree with Michalski (1991) when he states that the context of a concept is the set of concepts relevant to its intended meaning.

**FIGURE 5:**
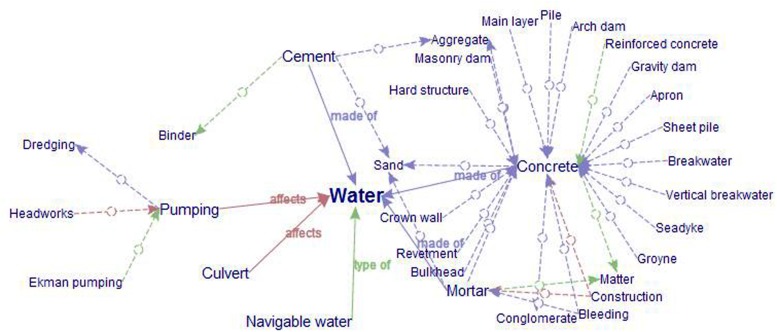
**WATER as recontextualized in Civil Engineering**.

The environmental domain was thus divided into a set of domain-based contexts (e.g., hydrology, geology, oceanography, civil engineering, etc.) and the relational power of concepts was constrained accordingly. This is done by assigning each conceptual proposition to one or more contextual domains based on a previously domain-based classified corpus. For example, the proposition CONCRETE *made_of* WATER only appears relevant in Civil Engineering texts, but not in a geological context. Thus, when constraints are applied, the network of WATER within the civil engineering sub-domain is recontextualized and becomes more meaningful (**Figure 5**).

Recontextualization is in line with Cruse’s (2002) approach to meaning (i.e., ways of seeing, microsenses, or context modulation) or Sperber and Wilson’s (1986) Relevance Theory, since semantic networks are dynamically built according to context salience. Thus, concepts themselves can also have their own situated nature. In this sense, Barsalou (2009, p. 1283) states that a concept produces a wide variety of situated conceptualizations that support goal achievement in specific contexts. In a similar way, this would be in consonance with semantic priming, which according to McNamara (2005) can be influenced by the context created by the types of semantic relations present in a test list.

## Conclusion and Future Work

In this paper, we have proposed a taxonomy of context primarily based on scope (local and global) and further divided into syntactic, semantic, and pragmatic facets for TKB design. Although context is a controversial notion interpreted and represented as needed in each field, we believe that for specialized knowledge representation in terminological resources, context should be much more than a textual excerpt.

Context modeling formally describes aspects of the linguistic, physical, and social world around us for purposes of understanding and communication. In this regard, it is necessary to determine what aspects to include and exclude from the model, and at what level of detail to model each of them.

Ideally, context specification and representation in specialized knowledge resources is conducive to the formulation of a common structure applicable to and valid for different languages and cultures based on a representational framework that allows for correspondences at different levels as well as for the inclusion of the syntactic, semantic and pragmatic features upon which this correspondence is based.

In EcoLexicon, various context parameters have been explored. Their specification has materialized in various modules representing different contextual aspects, ranging from term variation, collocations or knowledge-rich contexts, to dynamic conceptual networks, flexible definitional templates, or conceptually enriched graphical resources. However, much still remains to be done, especially with regards to the interdependence between all modules and the transition from local to global constraints. In the future, users will be provided with different types of information selected according to context. For this to be possible, context in all of its facets must be accounted for in a systematic and principled way.

## Author Contributions

All authors listed, have made substantial, direct and intellectual contribution to the work, and approved it for publication.

## Conflict of Interest Statement

The authors declare that the research was conducted in the absence of any commercial or financial relationships that could be construed as a potential conflict of interest.
